# Environmental pH controls antimicrobial production by human probiotic *Streptococcus salivarius*

**DOI:** 10.1128/jb.00059-25

**Published:** 2025-06-02

**Authors:** Dieu Linh Nguyen, Subhasree Saha, Aswin Thacharodi, Bharat Bhushan Singh, Sonali Mitra, Hackwon Do, Muthiah Kumaraswami

**Affiliations:** 1 Center for Molecular and Translational Human Infectious Diseases Research, Houston Methodist Research Institute 167626 , Houston, Texas, USA; 2 Department of Pathology and Genomic Medicine, Houston Methodist Hospital 735417 , Houston, Texas, USA; 3 Division of Life Sciences, Korea Polar Research Institute 123591, Incheon, Incheon, Korea; 4 Department of Polar Sciences, University of Science and Technology, Incheon, Republic of Korea; University of Illinois Chicago Pharmaceutical Sciences, Chicago, Illinois, USA

**Keywords:** gene regulation, pH sensing, probiotic, antimicrobial production, antimicrobial activity

## Abstract

*Streptococcus salivarius* K12 (SAL) is an oral probiotic used to treat or prevent oral infections caused by human pathogens. SAL produces at least three antimicrobials to exert its antimicrobial activity, namely, salivaricin A and salivaricin B, and the newly identified salivabactin. Salivabactin production is catalyzed by a polyketide/non-ribosomal peptide synthase hybrid biosynthetic gene cluster (BGC), termed as *sar-BGC*. The *sar-BGC* expression and salivabactin production are transient during SAL growth *in vitro* and *in vivo*, which may negatively impact SAL probiotic efficacy. To understand the molecular basis for transient *sar-BGC* expression, we assessed the impact of environmental pH on *sar-BGC* expression. We found that environmental acidification is a critical factor in promoting salivabactin antimicrobial activity and production by inducing *sar-BGC* expression. We further showed that acidic pH directly influences the quorum-sensing system that controls *sar-BGC* expression. During environmental acidification, SAL cytosol is acidified, which is sensed by a pH-sensitive histidine switch in the cytosolic transcription regulator, NrpR. The protonation of histidine during cytosolic acidification promotes high-affinity interactions between NrpR and its cognate intercellular signaling peptide, NIP, which leads to upregulation of *sar-BGC* expression. Collectively, our results indicate that SAL uses a sophisticated regulatory mechanism to orchestrate salivabactin production in an environment that is conducive to its antimicrobial activity.

**IMPORTANCE:**

Probiotic bacteria are important tools in combating bacterial infections. Probiotics exert their antimicrobial activity via several mechanisms, including antimicrobial production. However, discrepancies exist between the *in vitro* and *in vivo* efficacies of probiotics in inhibiting pathogen growth. Understanding the host and environmental factors that influence antimicrobial production and activity is critical for improving probiotic efficacy. In this study, we showed that the antimicrobial salivabactin produced by human oral probiotic *Streptococcus salivarius* K12 is active at acidic pH. We further elucidated the molecular mechanism by which *S. salivarius* coordinates salivabactin production in concert with environmental acidification, thereby maximizing salivabactin antimicrobial activity.

## INTRODUCTION

Probiotics are living microorganisms that confer health benefits to the host when consumed in adequate amounts (1). Probiotic bacteria exert antimicrobial activities by employing diverse molecular mechanisms including competing for nutrients and niches with the pathogens in the host, aiding host immune systems to facilitate pathogen clearance, and producing potent antimicrobials (1–6). Although the antimicrobial activity of probiotics is evident *in vitro*, the prophylactic or therapeutic efficacy of several probiotics *in vivo* is often inconsistent with their *in vitro* efficacy (1, 7, 8). One contributing factor to such inconsistencies is decreased production and activity of probiotic-derived antimicrobials *in vivo*. Thus, understanding the host and/or environmental factors controlling the probiotic-derived antimicrobial production and their antibacterial activity is critical to improve the probiotic efficacy *in vivo*.

*Streptococcus salivarius* strain K12 (SAL) was originally isolated from the oral cavity of a healthy infant (9) and has been used as a probiotic to prevent oral infections caused by human pathogens including *S. pyogenes*, also known as group A *streptococcus* (GAS) (10–12). SAL produces two lantibiotic peptides, salivaricin A (SalA) and salivaricin B (SalB), that are implicated in the *in vitro* antimicrobial activity of SAL (13–16). SAL also produces a third antimicrobial, salivabactin, that had potent antimicrobial activity *in vitro* against several gram-positive pathogens with a minimum inhibitory concentration (MIC) of 2 µg/mL (17). Furthermore, salivabactin was effective in treating intramuscular GAS infection in a mouse model of necrotizing myositis, indicating the *in vivo* therapeutic potency of salivabactin (17).

**Fig 1:**
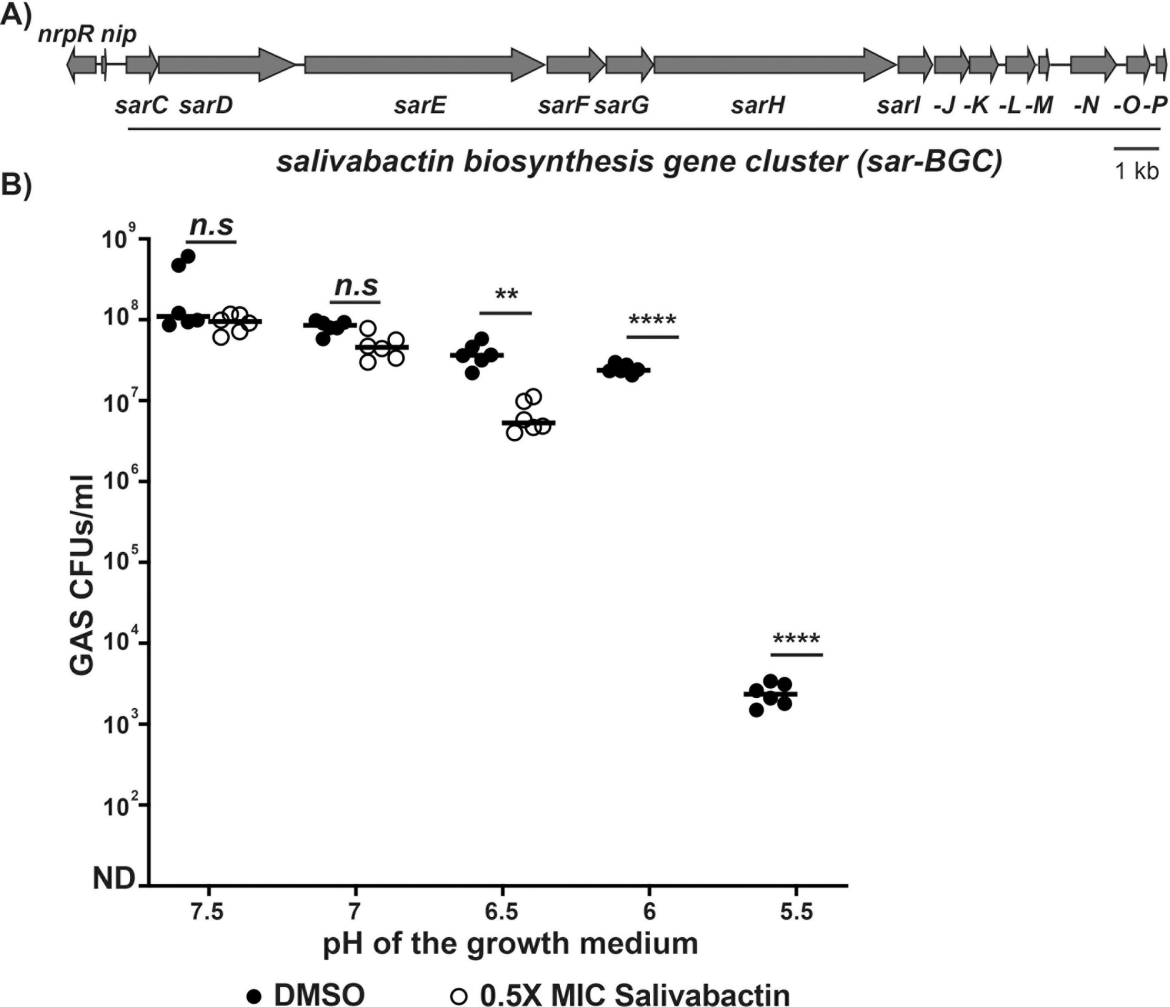
Acidic pH is optimal for salivabactin antimicrobial activity. (**A**) Genetic organization of the *sar* biosynthetic gene cluster (*sar-BGC*). The 14-gene operon encoding *sarC-P* genes is shown as arrows. The *sar-BGC* expression is controlled by a divergently transcribed leaderless communication peptide, NIP, and its cognate cytosolic receptor NrpR. (**B**) GAS (~10^5^ CFU/mL) was inoculated in THY broth adjusted to indicated pH. The GAS growth was supplemented with either carrier (DMSO, untreated) or 0.5× MIC of salivabactin, and inhibition of GAS growth was assessed by enumerating GAS colony-forming units (CFU/mL) after 16 h incubation. *P* values assessed by *t*-test are shown (*n.s*, not significant; **, *P* < 0.005; ****, *P* < 0.0001). ND indicates the limit of detection that was set at 10.

A 14-gene operon encoding a polyketide/non-ribosomal peptide synthase hybrid biosynthesis gene cluster (*PK/NRPS-BGC*), termed as *sar-BGC,* catalyzes the production of salivabactin (Fig. 1A). The expression of *sar-BGC* is controlled by a divergently transcribed quorum-sensing system that is composed of a leaderless communication peptide (LCP), NRPS-inducing peptide (NIP), and its cognate cytosolic receptor, NRPS regulator (NrpR) (Fig. 1A) (17, 18). NIP is expressed during high bacterial population density, secreted, reimported into the cytosol, and recognized by NrpR, which leads to upregulation of *sar-BGC*. Intriguingly, *sar-BGC* expression and salivabactin production were transient during SAL growth *in vitro* and in the host, which may compromise the probiotic efficacy of SAL (17). Thus, understanding the environmental factors and molecular basis for the transient *sar-BGC* expression may yield critical insights to sustain salivabactin production and improve probiotic efficacy.

In this study, we investigated the impact of environmental pH on the production and antimicrobial activity of salivabactin. We show that the antimicrobial activity of salivabactin is pH dependent and has maximal activity under acidic conditions. We further demonstrate that SAL employs a sophisticated pH-sensing mechanism in NrpR to orchestrate the upregulation of *sar-BGC* expression in concert with environmental acidification. We speculate that such convergence of pH and peptide sensing in NrpR aids the temporal production of salivabactin in an environment conducive to maximal antimicrobial activity.

## RESULTS

### Salivabactin antimicrobial activity is pH dependent

The production and antimicrobial activity of several characterized naturally occurring antimicrobials are pH sensitive, and their activity is maximal under either acidic or alkaline pH (19–24). To assess the interplay between pH and salivabactin activity, we compared the anti-GAS activity of salivabactin under different pH conditions. We used a subinhibitory concentration of salivabactin (0.5× MIC; ~1 µg/mL) to identify pH-specific differences in the antimicrobial activity (17). GAS reached high population density (>10^8^ CFU/mL) when grown in the absence of salivabactin but in the presence of carrier (DMSO) used to dissolve salivabactin (Fig. 1B). Salivabactin at subinhibitory concentration failed to affect GAS growth at near neutral or neutral pH conditions (pH 6.5–7.5). However, at pH 6.0, salivabactin had a potent anti-GAS activity as no viable GAS colonies were recovered in the salivabactin-treated group, whereas the analogous untreated group reached high population density (>10^7^ CFU/mL) (Fig. 1B). Although the untreated GAS had reduced growth at pH 5.5 compared to other pH conditions, salivabactin abolished GAS growth at pH 5.5 (1,000-fold reduction in CFU compared to untreated group) (Fig. 1B). These results indicate that salivabactin antimicrobial activity is maximal under slightly acidic pH conditions.

### *sar-BGC* expression occurs during environmental acidification

**Fig 2:**
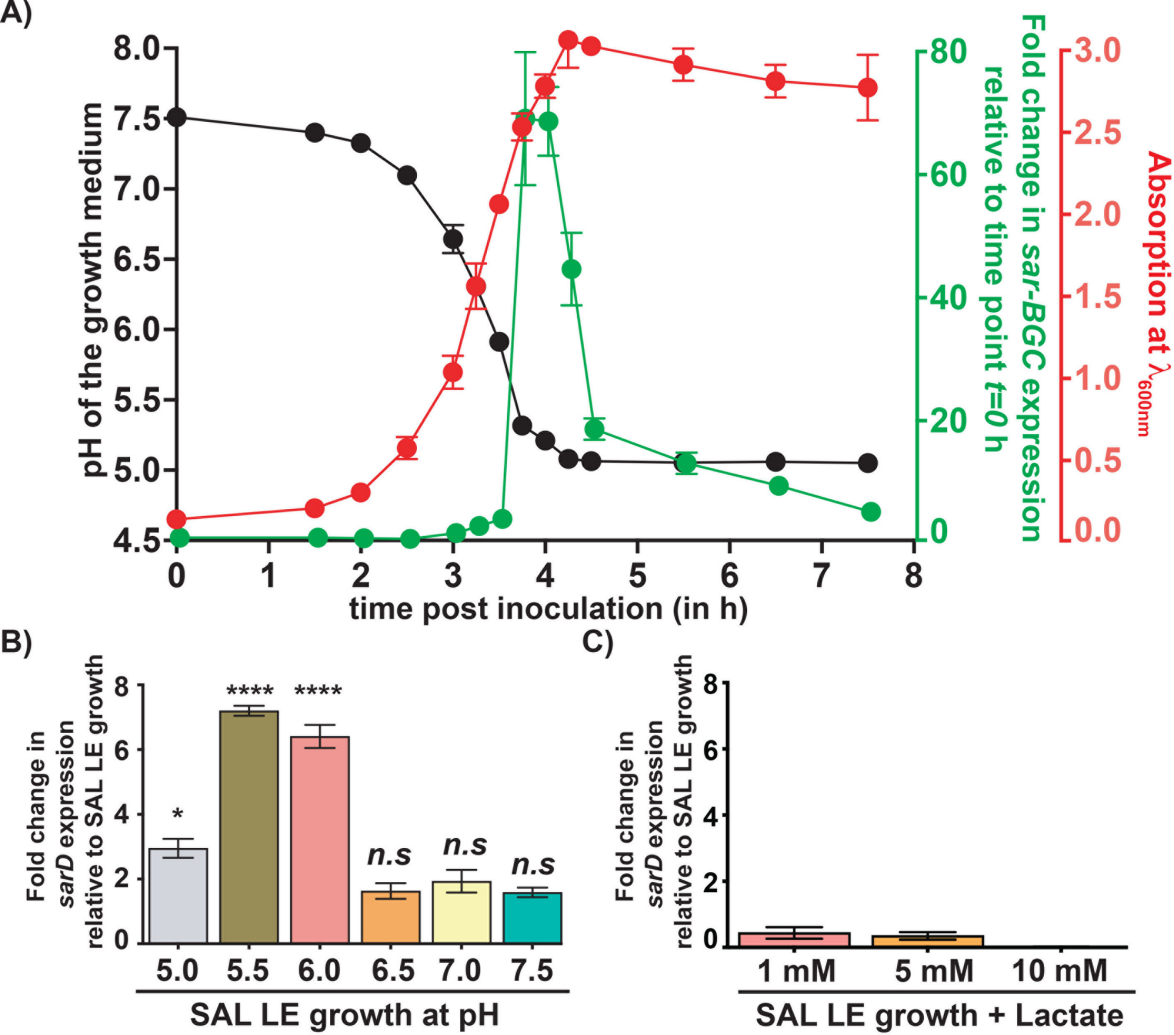
SAL upregulates *sar-BGC* expression in concert with environmental acidification. (**A**) Wild-type (WT) SAL was grown in THY broth, and samples were collected at the indicated time points. Growth medium pH, *sar-BGC (sarD*) transcript levels, and bacterial growth by absorption at wavelength 600 nm (*A*_600_) were determined. Right *y*-axes represent fold change in *sar-BGC* transcript levels (green) and *A*_600_ (red), whereas the left *y*-axis represents pH of the growth medium (black). Fold changes in transcript levels at indicated time points relative to starting culture (time point *t* = 0 h) are shown. Data are mean ± standard deviation for three biological replicates. (**B**) WT SAL grown in THY to late-exponential growth phase (LE, *A*_600_ ~ 2.0) was harvested by centrifugation, suspended in THY broth adjusted to indicated pH, and incubated for 15 minutes. The fold change in *sarD* transcript levels relative to WT-LE growth is shown. Data are mean ± standard deviation for three biological replicates. *P* values assessed by *t*-test analyses relative to WT LE growth are shown (*n.s*, not significant; *, *P* < 0.05; ****, *P* < 0.0001). (**C**) WT SAL grown in THY to late-exponential growth phase (LE, *A*_600_ ~ 2.0) was harvested by centrifugation, suspended in fresh THY broth supplemented with indicated final concentrations of sodium lactate, and incubated for 15 minutes. Fold change differences in *sarD* transcript levels relative to WT-LE growth are shown. Data are mean ± standard deviation for three biological replicates.

Since salivabactin antimicrobial activity is more pronounced under acidic pH (Fig. 1B) and lactic acid bacteria auto acidify their environment via lactic acid production, we hypothesized that SAL coordinates salivabactin production in concert with environmental acidification to potentiate its antimicrobial activity. To test this hypothesis, we correlated SAL growth kinetics *in vitro* to environmental pH changes in laboratory medium and *sar-BGC* expression. Consistent with the observations in other streptococci, SAL growth caused environmental acidification as the pH of the growth medium gradually decreased from near neutral (*t* = 0 h, pH 7.5) to acidic pH (*t* = 4.5 h, pH 5.0) in concert with increasing SAL growth (Fig. 2A). The *sar-BGC* expression was transient (3.5–4.5 h post inoculation; pH 5–5.5), and the onset of *sar-BGC* expression coincided with growth medium acidification. The initial induction of *sar-BGC* expression occurred during the late-exponential phase of growth (LE, *A*_600_ ~ 2.0) when the environmental pH was between 5 and 5.5 (*t* = 3.5 h, more than 60-fold induction) and reverted to basal levels as the pH of the growth medium dropped close to pH 5 (Fig. 2A). These observations indicate that SAL upregulates *sar-BGC* expression in concert with environmental acidification.

SAL and other lactic acid bacteria acidify their environment via lactic acid production during their growth. To distinguish whether lactic acid or environmental pH acts as a physiological signal that controls *sar-BGC* expression, we compared *sar-BGC* expression kinetics in SAL grown in pH-adjusted media. SAL grown to LE was harvested and resuspended in laboratory medium adjusted to different pH. After 15 min incubation, cells were collected and *sarD* transcript levels in each pH were assessed by quantitative real-time PCR (qRT-PCR). The pH alterations had no effect on bacterial viability (data not shown). However, the environmental pH influenced *sar-BGC* expression as transcript levels of *sar-BGC* were significantly increased at pH values 5.5 and 6.0 compared to LE growth, whereas no significant alterations in *sar-BGC* expression were observed at pH either below 5.5 or above 6.0 (Fig. 2B). To test whether lactate acts as a signal for the induction of *sar-BGC* expression, we assessed *sarD* transcript levels in SAL grown in the presence of lactate. However, lactate supplementation did not induce *sar-BGC* expression, and *sarD* transcript levels in lactate-supplemented SAL were comparable to those of SAL LE growth (Fig. 2C), indicating that lactate does not control *sar-BGC* expression. These results indicate that environmental acidification is the physiological cue that controls *sar-BGC* expression.

### Environmental pH controls *sar-BGC* expression by influencing NIP signaling pathway

**Fig 3:**
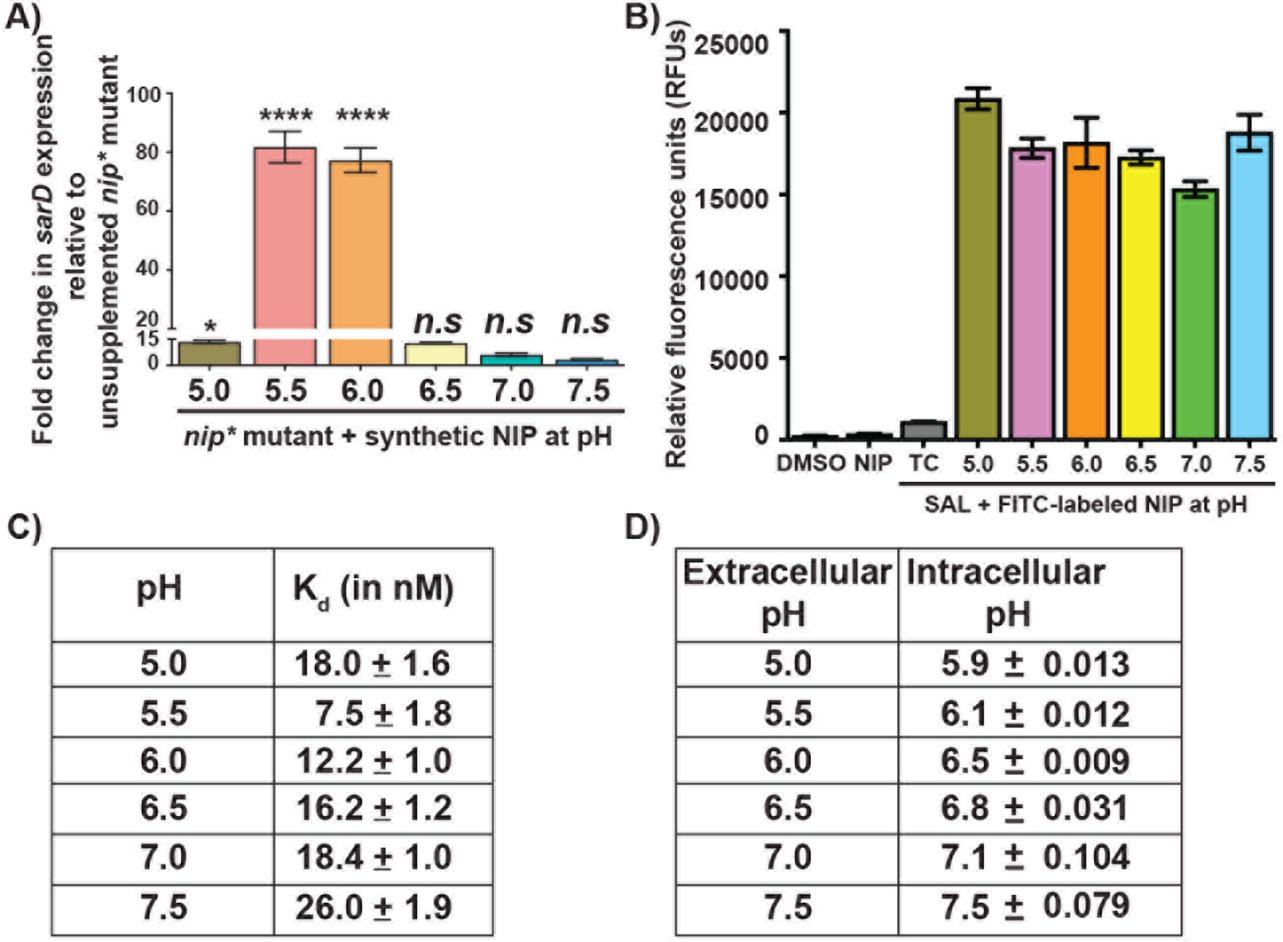
Environmental acidification activates the NIP signaling pathway by influencing NIP-NrpR interactions. (**A**) The nip* mutant grown in THY to late-exponential growth phase (LE ~ 2.0) was harvested by centrifugation, suspended in fresh THY adjusted to indicated pH, supplemented with 100 nM synthetic NIP, and incubated for 30 minutes. The *sarD* transcript levels relative to unsupplemented nip* mutant are normalized to 1 and used as a reference to determine the fold changes in *sarD* levels in synthetic NIP-supplemented samples. *P* values assessed by Kruskal-Wallis analyses relative to unsupplemented nip* mutant are shown (*n.s*, not significant; *, *P* < 0.05; ****, *P* < 0.0001). (**B**) Environmental acidification does not affect the reimport of exogenously added synthetic FITC-NIP into the cytosol. The nip* mutant grown to late-exponential phase (*A*_600_ ~ 2.0) was harvested by centrifugation and resuspended in pH-adjusted THY broth. Cells were supplemented with either unlabeled NIP (NIP) or FITC-labeled NIP. Cells supplemented with the carrier for the synthetic peptide (DMSO) were used as a control. After 30 minutes of incubation, cells were harvested, washed, and resuspended in PBS, and cytosolic fluorescence in the clarified cell lysates was assessed using excitation and emission wavelengths of 480 and 520 nm, respectively. Cells incubated with FITC-NIP but not lysed (TC, total cells) were used to determine the fluorescence associated with cell surface attached FITC-NIP. Fluorescence in unlysed total cells (TC) The changes in relative fluorescence units (RFU) relative to the unsupplemented nip* mutant are shown. (**C**) The NrpR-NIP binding constants in binding buffer adjusted to indicated pH. (**D**) The calculated SAL cytosolic pH values in the tested extracellular pH were determined by the equation derived from the calibration curve.

The *sar-BGC* expression is primarily controlled by a leaderless communication peptide (LCP) pathway that is composed of a secreted intercellular signal, leaderless peptide NIP, and its cognate cytosolic receptor, NrpR. Thus, we hypothesized that intercellular signaling by NIP is pH sensitive and that upregulation of *sar-BGC* expression during environmental acidification occurs via the NIP signaling pathway. To test this hypothesis, we performed synthetic NIP addition experiments under different pH conditions using a *nip*-inactivated nip* mutant in which NIP production is disrupted by replacing the start codon (ATG) with stop codon (TAG). The *sar-BGC* expression is abolished in the nip* mutant, and induction of *sar-BGC* expression in the nip* mutant depends exclusively on the exogenous addition of synthetic NIP. Cells were grown to the LE phase of growth, harvested, and resuspended in fresh laboratory medium adjusted to different pH. Cells were supplemented with synthetic NIP, and the effect of pH on NIP-dependent activation of *sar-BGC* expression was assessed by qRT-PCR. The addition of NIP failed to cause significant induction of *sar-BGC* expression in the nip* mutant under environmental pH between 6.5 and 7.5 as well as at pH 5.0 (Fig. 3A). However, significant NIP-dependent upregulation of *sar-BGC* was observed at pH 5.5 or 6.0 compared to unsupplemented SAL growth (more than 75-fold induction) (Fig. 3A), indicating that environmental pH influences *sar-BGC* expression by modulating the regulatory activity of the NIP signaling pathway.

### Environmental pH does not impact NIP reimport

NIP signaling is a multi-step process as it involves secretion and reimport of NIP into the cytosol, NIP recognition by the cytosolic receptor NrpR, and activation of *sar-BGC* expression by NIP-bound NrpR. Since the regulatory activity of exogenously added NIP was affected by environmental pH, we reasoned that pH influences NIP signaling pathway by either affecting NIP reimport and/or cytosolic NIP recognition by NrpR. To distinguish these two possibilities, we first assessed the effect of environmental pH on NIP reimport by monitoring the cytosolic fluorescence of exogenously added fluorescein isothiocyanate (FITC)-labeled NIP. Cells grown to LE were resuspended in pH-adjusted media and incubated for 15 min with FITC-NIP. Subsequently, cells were washed, lysed, and cytosolic NIP levels were assessed by measuring the FITC-NIP associated fluorescence in the clarified cell lysates. No significant differences in cytosolic fluorescence were observed in all the tested pH conditions (Fig. 3B). To determine whether the observed fluorescence in clarified cell lysates represents cytosolic FITC-NIP, not cell surface-associated FITC-NIP, we performed a similar FITC-NIP addition experiment and measured fluorescence in total cells without lysis. No significant increase in fluorescence was observed compared to that of carrier- or unlabeled NIP-supplemented samples (Fig. 3B), indicating that observed fluorescence in lysed samples is associated with cytosolic FITC-NIP. Together, these results demonstrate that environmental pH does not affect the internalization of exogenously added NIP into the cytosol.

### Environmental pH affects *sar-BGC* expression by influencing NIP-NrpR interactions

To investigate the effect of pH on NIP and NrpR interactions, we measured the binding affinities of NrpR for NIP by fluorescence polarization (FP) assay. Our results show that NrpR binds to NIP with higher affinity at pH values 5.5 and 6.0 compared to their interactions at either near neutral pH (pH ≥ 6.5) or at pH 5.0. The affinity of NrpR for NIP decreased by more than twofold at near neutral pH or pH 5.0 compared to the binding affinity at pH 5.5 (Fig. 3C; Fig. S1). These results demonstrate that environmental acidification affects cytosolic NIP recognition by NrpR, which leads to alterations in *sar-BGC* expression.

### Environmental acidification influences SAL cytosolic pH

Given that the optimal high-affinity NIP-NrpR interactions require acidified cytosol, we assessed the impact of environmental acidification on SAL cytosolic pH by measuring SAL intracellular pH during environmental pH alterations. We used the pH-dependent fluorescence of fluorophore 6-carboxyfluorescein succinimidyl ester (cFSE) to measure the cytosolic pH of SAL incubated under different pH conditions. The diacetate form of cFSE precursor, cFDASE, is cell permeant and imported into the cell. Upon internalization, the cytosolic cFDASE is hydrolyzed to fluorescent cFSE in the cytosol, which forms a stable conjugate with the intracellular proteins and prevents the leakage of cFSE from the cells. The relative ratios of fluorescence intensities in the clarified cell lysates between pH-sensitive (490 nm) and pH-insensitive (435 nm) excitation wavelengths can be used to determine intracellular pH.

SAL was grown to late-exponential phase (*A*_600_ ~ 2.0) and incubated with cFDASE. The cFSE-loaded cells were washed and suspended individually in buffers adjusted for indicated pH. After brief incubation, fluorescence intensities were measured. Our results demonstrated that SAL intracellular pH decreases in response to environmental acidification. At neutral (pH 7.0) or near neutral pH (pH 7.5), the environmental pH did not impact SAL cytosolic pH as both extracellular and intracellular pH values remained similar (Fig. 3D). However, under below neutral pH conditions (pH 5.0–6.5), the intracellular pH decreased, and SAL maintained a pH difference (ΔpH = pH_intracellular_ − pH_extracellular_) of 0.3–0.9 units with higher differences occurring under a more acidified environment (Fig. 3D). These results indicate that when environmental acidification occurs (pH 5.0–6.0), the intracellular pH (pH ~ 5.9–6.5) becomes conducive for high-affinity NIP-NrpR interactions and upregulation of *sar-BGC* expression.

### Molecular mechanism of pH sensing by NrpR

**Fig 4:**
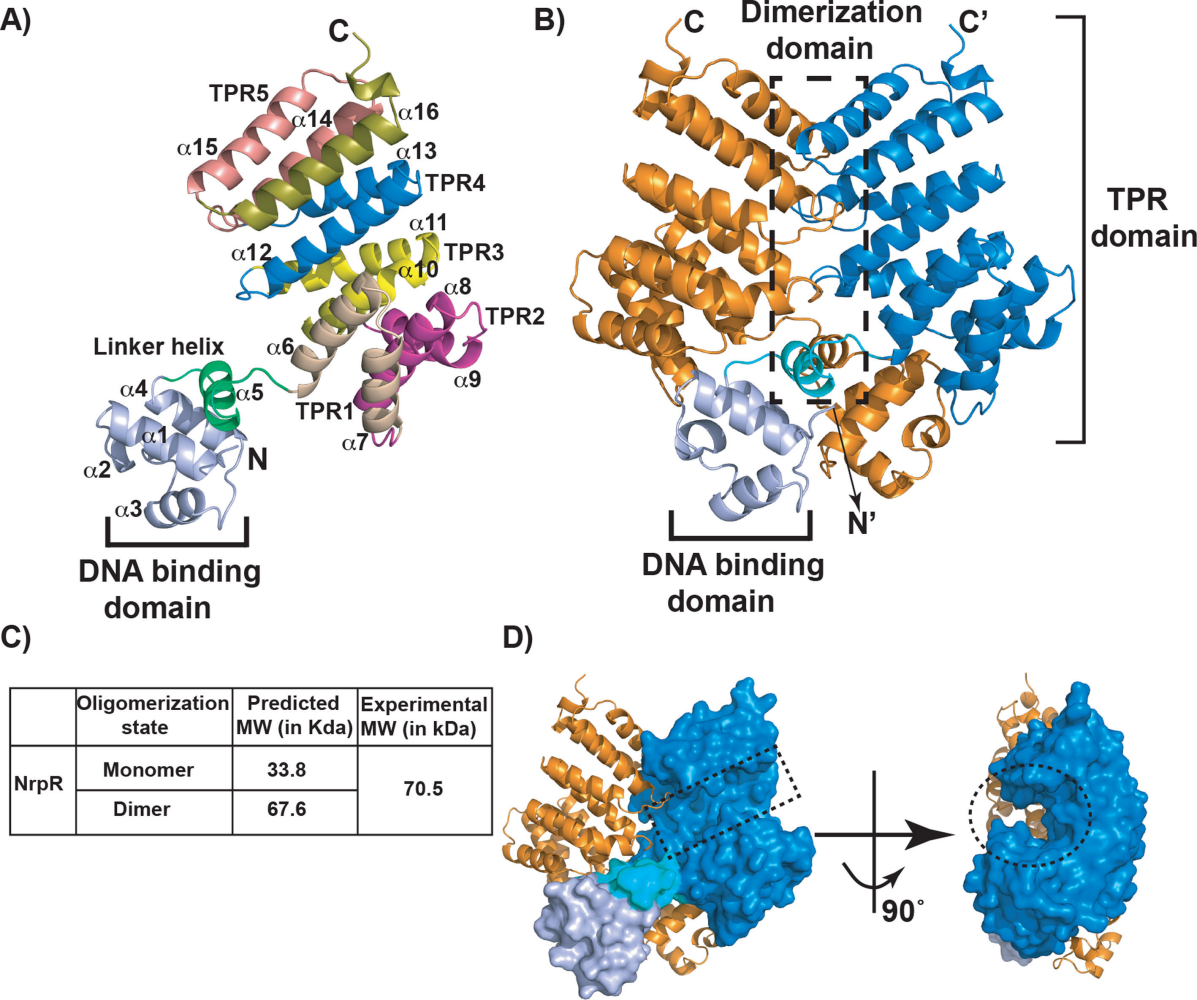
Crystal structure of NrpR. (**A**) The crystal structure of an NrpR subunit. The DNA-binding domain (**A1-A4**) at the amino-terminal domain is colored purple and labeled, and the linker α-helix (**A5**) connecting the DNA-binding domain and C-terminal domain is shown in green. Each tetratricopeptide repeat motif (TPR1–TPR5; **A6-A16**) in the C-terminal domain is color-coded and labeled. The N- and C-terminus of NrpR subunit is labeled N and C, respectively. (**B**) The dimeric structure of NrpR. Each subunit is colored blue and orange, and the DNA-binding and putative peptide-binding TPR domains in one subunit are labeled. The dimerization interface between the two subunits is highlighted by dashed lines. The N- and C-terminus of one subunit is marked as N and C, respectively. (**C**) The oligomerization state of NrpR as assessed by size exclusion chromatography. A table showing the theoretical molecular weight of various oligomeric states of NrpR and the experimentally calculated molecular weight of NrpR based on the elution profile in a Superdex 200 size exclusion chromatography is shown. (**D**) Surface representation of the crystal structure of NrpR dimer. One subunit of NrpR dimer is shown as ribbons, and the second subunit is depicted in surface representation. The concave surface in the C-terminal TPR domain containing the putative peptide-binding pocket of NrpR is marked in dashed lines.

To elucidate the molecular mechanism of pH sensing by NrpR, we determined the crystal structure of cytosolic receptor NrpR (amino acids 1–285) to 2.6 Å resolution (Table 1), the first full-length structure of an LCP receptor (Fig. 4) (18, 25, 26). The crystal belonged to the *I*2_1_ space group, and each asymmetric unit has an NrpR dimer (Fig. 4B). NrpR formed a crystallographic dimer. Consistent with this observation, size exclusion chromatography analyses of purified recombinant NrpR showed that NrpR exists as a dimer in solution with a calculated molecular weight of 70.5 kDa (Fig. 4C; Fig. S2). The overall architecture of NrpR is similar to the DNA-binding members of the RRNPPA family of regulators. Each NrpR subunit is composed of 17 α-helices and has two distinct functional domains: an amino-terminal DNA-binding domain (helices α1–α4) with a classical helix-turn-helix (HTH) DNA-binding motif and a C-terminal helical domain (CTD) (helices α6–α17) that has the characteristic tetratricopeptide repeat (TPR) motif-containing domain (Fig. 4A) (25, 27). The two domains are connected by a linker helix α5 (Fig. 4A). The NrpR-CTD has five TPR motifs (TPR1–TPR5) and each TPR motif is made of a pair of antiparallel a-helices (Fig. 4A). The TPR motifs form a super-helical structure in the CTD with an exposed concave surface (Fig. 4D). Comparison of the NrpR structure with its structural homologs RopB from *S. pyogenes* (PDB: 5DL2; sequence identity, 52%) (28) and Rgg2 from *S. dysgalactiae* (PDB: 5W4M; sequence identity, 23.9%) (29) suggests that the concave surface in the CTD of NrpR constitutes the NIP-binding pocket (Fig. 4D). The putative NIP-binding pocket of NrpR is lined by the helices α6, α10, α12, α14, and α15. NrpR and RopB share critical residues essential for protein-peptide interactions, including peptide backbone-contacting conserved asparagines N152 and N192 on the floor of the peptide-binding pocket, suggesting that NrpR employs analogous amino acids to interact with NIP.

**TABLE 1:** X-ray data collection and refinement statistics

Data set	NrpR
X-ray source	PLS BL-5C beamline
Space group	*I*2_1_
Unit-cell parameters (Å, °)	*a* = 34.75, *b* = 128.26, *c* = 72.63, α = γ = 90.0, β = 100.6
Wavelength (Å)	0.9794
Resolution (Å)	36.68–2.59 (2.71–2.59)
Total reflections	23,974 (2,958)
Unique reflections	56,903 (2,767)
Average *I*/σ (*I*)	1.85 (2.58)
*R*_merge_^*a*^	0.030 (0.27)
CC1/2	0.999 (0.952)
Redundancy	2.5 (2.6)
Completeness (%)	97.2 (97.7)
Refinement	
Resolution range (Å)	36.71–2.59
No. of working set reflections	9,016 (675)
No. of test set reflections	458 (20)
No. of amino acid residues	285
No. of conformers	1
*R*_cryst_^*b*^	0.18 (0.27)
*R*_free_^*c*^	0.19 (0.28)
r.m.s. bond length (Å)	0.007
r.m.s. bond angle (°)	1.49
Average *B* value (Å^2^) (protein)	81.64
Average *B* value (Å^2^) (solvent)	71.02
Ramachandran plot	
Favored (%)	92.23
Allowed (%)	5.65
Outliers (%)	2.21

^*a*^ *R*_merge_ = ∑｜<*I*> − *I*｜/∑<*I*> .

^*b*^ *R*_cryst_ = ∑｜|*F*_o_| − |*F*_c_|｜/∑|*F*_o_|.

^*c*^ *R*_free_ calculated with 5% of all reflections excluded from refinement stages using high-resolution data.

### A histidine below the NIP-binding pocket is critical for pH sensing by NrpR

**Fig 5:**
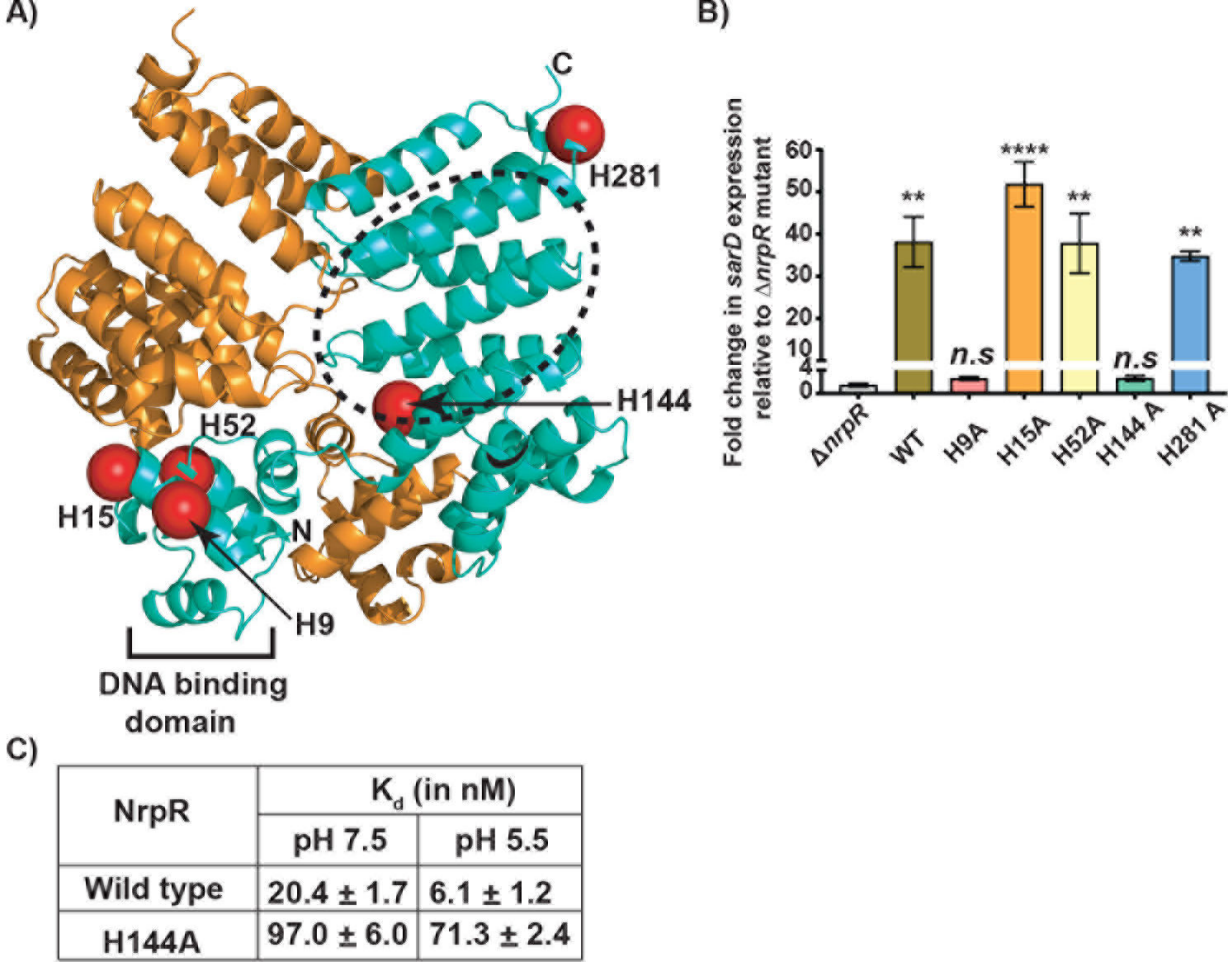
Histidine 144 (H144) is critical for pH sensing by NrpR. (**A**) The location of histidines in the NrpR dimer is shown as red spheres and labeled. The DNA-binding domain is marked and labeled, whereas the putative peptide-binding pocket of NrpR is highlighted in dashed lines. The N- and C-terminus of one subunit is marked as N and C, respectively. (**B**) Analysis of the *sarD* transcript levels in *nrpR* mutant strains containing single alanine substitution at histidines. The *sarD* expression in the *∆nrpR* mutant was used as a reference, and fold changes in *sarD* transcript levels relative to the reference are shown. Data are mean ± standard deviation for three biological replicates. *P* values (**P < 0.01 and ****P < 0.0001; *n.s*, not significant) as assessed by Kruskal-Wallis analyses are shown. (**C**) NrpR–synthetic NIP-binding constants for the indicated recombinant NrpR mutant proteins as assessed by FP assay are shown.

The peptide binding and gene regulatory activity of NrpR is favored between pH 5.5 and 6.0, which is near the p*K*_a_ value of free histidine side chain (p*K*_a_~ 6.0). At pH 6.0, the histidine side chain becomes protonated and is shown to participate in acidic pH sensing and contribute to the acidic pH-dependent gene regulation of several bacterial proteins (28, 30–35). Thus, we hypothesized that the histidines in NrpR are involved in pH sensing and *sar-BGC* upregulation by NrpR. The NrpR has a total of five histidines that include H9, H15, H52, H144, and H281. Mapping the histidines on the NrpR structure showed that H9 and H15 are located within the N-terminal DNA-binding domain, H52 is situated between the N-terminal domain and linker helix, and H144 and H281 are present in the C-terminal domain (Fig. 5A).

To deduce the role of histidines in pH-dependent gene regulation by NrpR, we generated isoallelic SAL mutant strains containing single alanine substitution at each histidine and assessed the mutant strains for *sar-BGC* expression by qRT-PCR. The *sar-BGC* transcript levels in H15A, H52A, and H281A mutant strains were comparable to that of WT, indicating that these three histidines are not involved in pH sensing (Fig. 5B). However, alanine substitution at either H9 or H144 abolished *sar-BGC* expression, and transcript levels were similar to that of the ∆*nrpR* mutant (Fig. 5B). Since H9 is located in the DNA-binding domain, it is likely that the observed phenotype is due to its role in DNA binding and unlikely to have a role in NIP binding by NrpR. Contrary to this, the side chain of H144 is located at the bottom of the predicted NIP-binding pocket of NrpR and positioned to influence NrpR-NIP interactions in response to pH alterations (Fig. 5A). Since environmental pH affects NrpR-NIP interactions, we next compared the pH-dependent high-affinity interactions of WT NrpR and H144A with NIP by FP assay. Consistent with previous observations, the affinity of WT NrpR to NIP increased at pH 5.5 compared to neutral pH (Fig. 5C; Fig. S3). Conversely, the recombinant H144A mutant protein remained insensitive to pH alterations as H144A engaged in low-affinity interactions with NIP at both pH values 5.5 and 7.5 (Fig. 5C). These results demonstrate that H144, located beneath the NIP-binding pocket of NrpR, is responsible for pH sensing and pH-dependent alterations in NIP binding and *sar-BGC* regulation by NrpR.

## DISCUSSION

Our results demonstrate that the antimicrobial activity of probiotic SAL-derived salivabactin is pH dependent, with slightly acidic pH favoring maximal inhibitory activity (Fig. 1B). We further show that, to accommodate the pH requirements of salivabactin for its antimicrobial activity, SAL employs a sophisticated pH-sensing regulatory circuit that ensures timely production of salivabactin in an acidified environment. SAL uses a pH-sensing histidine switch in transcription regulator NrpR to sense the acidified environment and activate *sar-BGC* expression (Fig. 5). The acidic pH likely induces protonation of histidine, which promotes high-affinity interactions between NrpR and its cognate ligand, NIP, upregulation of *nip* and *sar-BGC* expression, and salivabactin production.

Environmental pH has been shown to be a critical factor in the production and activity of several naturally occurring antimicrobials (19–24, 36). Several lactic acid bacteria-derived antimicrobials are produced at the highest level at extracellular pH near 5.5 (19–24, 36). However, for most of these systems, the molecular mechanisms by which the antimicrobial producers sense environmental pH and coordinate antimicrobial production in concert with extracellular pH changes remain unknown (19, 21, 36). Our findings demonstrate that SAL employs a pH-sensing histidine switch in the regulatory circuit that controls *sar-BGC* expression. When environmental pH decreases, the acidified SAL cytosol is monitored by the side chain of H144 in NrpR, which triggers allosteric changes in NrpR, aids high-affinity recognition of NIP, and causes the upregulation of *sar-BGC*. Thus, it is likely that similar histidine-based pH sensors may exist in the regulatory elements controlling the production of lactic acid bacteria-derived antimicrobials and participate in the coordination of antimicrobial production in concert with environmental pH.

Our genetic and biochemical results show that the side chain of H144 is critical for pH-dependent induction of NIP recognition by NrpR and *sar-BGC* expression. Since NrpR was crystallized under neutral pH conditions, the crystal structure failed to divulge the pH-dependent allosteric changes in NrpR that lead to transcription activation of *sar-BGC*. Interestingly, RopB, the closest structural homolog of NrpR from GAS, also uses a histidine (H144) as a pH sensor to coordinate the timely production of the virulence factor, secreted protease SpeB. Analyses of RopB revealed that the side chain of H144 becomes protonated during environmental acidification, and the protonated H144 is engaged in hydrogen bonding interactions with the side chains of Y176, Y182, and E185 located beneath the peptide-binding pocket of RopB (28). It was proposed that such interactions allosterically facilitate the access of the peptide-binding pocket and promote high-affinity interactions between RopB and its cognate peptide, SIP. A closer look at the immediate vicinity of H144 in the NrpR structure showed that the side chains of Y176, Y182, and E185 in RopB are conserved in NrpR. However, only the side chains of H144, Y176, and E185 are present in similar locations in the structures of RopB and NrpR, whereas Y182 in NrpR occupies a position dissimilar to that of RopB (Fig. S4). Thus, it is likely that NrpR may employ a similar molecular mechanism of pH sensing and signal transduction during environmental acidification and orchestrate the upregulation of *sar-BGC*. Structures of NrpR crystallized under acidic pH may provide the molecular details of pH-induced hydrogen bonding network by H144 and ensuing allosteric changes in NrpR that lead to increased salivabactin production during acidic environmental pH.

In summary, our results show that environmental pH is a critical factor in the regulation of *sar-BGC* expression, salivabactin production, and salivabactin activity. The acidic pH acts upstream of the peptide-dependent quorum-sensing pathway and influences its gene regulatory activity by affecting receptor-peptide interactions. Furthermore, our findings suggest potential pH modifications to probiotic formulations, which may improve the antimicrobial activity and probiotic efficacy of SAL.

## MATERIALS AND METHODS

### Bacterial strains, plasmids, and growth conditions

*Streptococcus salivarius* K12 (SAL) was cultured on a trypticase soy agar supplemented with 5% sheep blood (BSA; Becton Dickinson) or in a Todd–Hewitt broth with 0.2% (wt/vol) yeast extract (THY; DIFCO). All SAL growth experiments were performed in duplicate on three separate occasions, totaling six replicates. Overnight cultures were diluted into fresh media to achieve an initial optical density at 600 nm (*A*_600_) of 0.05, and bacterial growth was monitored by measuring the absorbance at 600 nm. *Escherichia coli* DH5α strain and BL21(DE3) strain was used for plasmid construction and recombinant protein overexpression, respectively. For protein overexpression, the *E. coli* strain was grown in lysogeny broth (LB broth; Fisher). Kanamycin was added to the media at final concentrations of 50 µg/mL. Strains and plasmids used in the study are listed in Table S1.

### Salivabactin antimicrobial activity assay

The pH dependence of anti-GAS activity of salivabactin was determined by broth microdilution according to the Clinical and Laboratory Standards Institute guidelines. GAS cultures grown to exponential phase of growth were diluted to approximately 5 × 10^4^ CFU/mL in THY broth adjusted to indicated pH with HCl. The diluted inoculum (300 µL) was placed in 96-well plates, and salivabactin was added to each well to a final concentration of 1 µg/mL (2 µL; 0.5× MIC). After 16–24 h incubation at 37˚C, the viability of GAS was determined by enumerating the colony-forming units (CFU) per milliliter. MIC assays were performed in biological triplicates.

### Correlation of SAL growth kinetics with *sar-BGC* expression and growth medium pH

The overnight SAL growth was diluted 1:100 with fresh THY and grown to indicated phases of SAL growth. Samples were collected at indicated time points, and bacterial growth, the pH of growth medium, and *sar-BGC* expression were measured. Bacterial growth was determined by absorbance measurements at 600 nm, whereas *sar-BGC* transcript levels were assessed by quantitative real-time PCR (qRT-PCR).

### Transcript level analysis by qRT-PCR

SAL strains grown to the indicated time points were collected and treated with two volumes of RNAprotect (Qiagen) for 10 minutes at room temperature. Cells were harvested by centrifugation at 4,000 rpm for 10 minutes, and cell pellets were stored at −80˚C till use. Cells were thawed and lysed by fast prep using beads (MP Biomedicals). RNA was isolated and purified using the RNeasy kit (Qiagen). Purified total RNA was analyzed for quality and yield by Agilent Bioanalyzer. Following quality control, cDNA was synthesized from the purified RNA using Superscript III (Invitrogen). Transcript levels were measured by qRT-PCR with the ABI 7500 Fast System (Applied Biosystems). Using the transcript levels of housekeeping gene *tufA* as reference, we compared *sar-BGC* transcript levels using the ΔCT method. The primers utilized for qRT-PCR are listed in Table S2.

### Synthetic peptide addition assay

Synthetic peptides (>95% purity) were obtained as lyophilized powder from Peptide 2.0 (Chantilly, VA). The peptides were suspended in 100% DMSO to a concentration of 10 mM, aliquoted, and stored at −20°C. Working stocks were prepared by diluting the aliquots fresh in 25% DMSO for each experiment.

### Peptide reimport studies by fluorescence measurements

To demonstrate the cytosolic internalization of exogenously added FITC-labeled LCP_ss_, we grew indicated the *S. salivarius* strains to late-exponential phase of growth (*A*_600_ ~2.0) and incubated them with either the indicated synthetic peptide or the carrier for the synthetic peptides (DMSO) for 30 min at 37°C. Cells were harvested by centrifugation, washed three times with sterile PBS, and resuspended in equal volume of PBS. Cells were lysed by FastPrep-24 (MP Biomedicals), and lysates were clarified by centrifugation at 13,000 rpm at 4°C for 30 min. Samples were analyzed in 100 µL volume using excitation and emission wavelengths of 490 and 520 nm, respectively. Readings were taken using a Biotek microplate reader (Biotek) and fluorescence measurements in relative fluorescence units were reported.

### Cloning, expression, and purification of recombinant NrpR

The coding region of NrpR (amino acids 1–285) was cloned into pET-28a(+) plasmid (Novagen, Madison, WI, USA) at the NdeI restriction enzyme site to generate N-terminal hexa-histidine tagged recombinant NrpR. The plasmid was introduced into BL21 (DE3) and used for protein overexpression. Cells were grown in lysogeny broth (LB) containing 50 µg/mL kanamycin to *A*_600_ ~ 0.5, and protein overexpression was induced by the addition of 1 mM isopropyl β-d-1-thiogalactopyranoside (IPTG) for 2 days at 15°C. Cells were harvested by centrifugation at 6,000 rpm for 20 min. The cells were resuspended in buffer A (20 mM Tris-HCl, pH 8.5, 200 mM NaCl, 1 mM TCEP, and 10% glycerol), disrupted by sonication, and cell lysates were isolated by centrifugation at 16,000 rpm for 40 min at 4°C. The clarified cell lysate was loaded onto a Ni-NTA agarose column (Qiagen, Hilden, Germany), and bound protein was eluted with buffer A containing 300 mM imidazole. The eluted fractions containing hexa-histidine tagged NrpR were pooled, concentrated, and treated with thrombin (1 unit per milligram of NrpR) (Sigma-Aldrich, Missouri, USA) to remove the hexa-histidine residues. The untagged NrpR was further purified by size exclusion chromatography using a Superdex 200 column (GE Healthcare, Piscataway, NJ, USA). The purified NrpR was concentrated to 15 mg/mL in storage buffer (20 mM Tris-HCl, pH 8.5, 200 mM NaCl, 1 mM TCEP, and 5% glycerol) using an Amicon YM-30 concentrator (Merck, Darmstadt, Germany).

### Fluorescence polarization (FP) assay

Synthetic NIP-NrpR interactions were analyzed by FP assay as described previously (26, 28). The polarization (*P*) of fluorescein-labeled synthetic NIP increases as a function of protein binding. The resulting plot correlating the millipolarization (*P* × 10^–3^) against protein concentration was used to determine the equilibrium dissociation constants. fluorescein-labeled NIP (1 nM) in binding buffer (20 mM potassium phosphate, pH 6.0, 75 mM NaCl, 2% DMSO, 1 mM EDTA, and 0.0005% Tween 20) was titrated against increasing concentrations of purified NrpR, and the resulting change in polarization was measured. Samples were excited at 490 nm and emission measured at 530 nm. All data were plotted using KaleidaGraph, and the resulting plots were fitted with the equation *P* = {(*P*_bound_ − *P*_free_)[protein]/(*K*_D_ + [protein])} + *P*_free_, where *P* is the polarization measured at a given protein concentration, *P*_free_ is the initial polarization of the free ligand, *P*_bound_ is the maximum polarization of specifically bound ligand, and [protein] is the protein concentration. Nonlinear least squares analysis was used to determine *P*_bound_ and *K*_d_. The binding constant reported is the average value from at least three independent experimental measurements.

### Measurement of intracellular pH

The cytosolic pH (pHi) was measured using a fluorescent probe method described previously (28, 37, 38). Cells were cultured to the mid-exponential phase of SAL growth (*A*_600_ ~ 1.0) in THY, collected by centrifugation, washed twice with 150 mM NaCl, and resuspended in 50 mM HEPES buffer (pH 8.0). Cells were subsequently incubated at 37°C for 20 min with 10 µM carboxyfluorescein diacetate succinimidyl ester (cFDASE, Invitrogen). cFDASE is hydrolyzed to carboxyfluorescein succinimidyl ester (cFSE) in the bacterial cytosol, which then binds to aliphatic amines on intracellular proteins. After incubation, cells were washed and resuspended in 50 mM potassium phosphate buffer (pH 7.5). Cells were treated with 10 mM glucose for 30 min at 30°C to remove unbound cFSE and washed twice with and resuspended in 150 mM NaCl. An equal volume of cFSE-labeled cells was suspended in 0.5 mL of buffer with different pH. After 5 min incubation, fluorescence intensities were measured using excitation wavelengths of 490 nm (pH-sensitive) and 435 nm (pH-insensitive) and emission wavelength at 520 nm. The ratio of emission from excitation at 490 nm versus 435 nm was calculated for both cell suspensions (C) and filtrates (F) as R490/435 = (C490 − F490)/(C435 − F435). A calibration curve was established using potassium phosphate buffers with pH values ranging from 5.5 to 8.0, and a cubic equation was derived from this ratio value. The intracellular pH values for SAL were then determined using this cubic equation from the calibration curve.

### Crystallization, data collection, and structure determination

Crystallization trials using purified NrpR were performed at 20°C using a mosquito high-throughput crystallization robot (TTP Labtech, UK). The diffracting crystals of NrpR were obtained in 0.7 M sodium citrate tribasic dihydrate and 0.1 M Bis-Tris propane, pH 7.0. X-ray diffraction data sets were collected using paratone as a cryo-protectant on the 5C beamline at Pohang Light Source (PLS) operated by the Pohang Accelerator Laboratory (PAL; Pohang, Republic of Korea). Diffraction data using NrpR crystals were collected to a final resolution of 2.59 Å and processed with HKL-2000 (39). The NrpR crystal belonged to the *I2_1_* space group, with unit-cell parameters *a* = 34.75, *b* = 128.26, *c* = 72.63 Å, α = γ = 90, and β = 100.6. The data set was indexed and integrated using the XDS software (40) and scaled using Aimless from the CCP4 package (41). After molecular replacement using the RopB structure as the search model (PDB code: 5DL2), the coordinates were refined using the combination of the following programs: Coot (42), Refmac5 (43), and Phenix (44). The X-ray data collection and refinement are listed in Table 1.

### Size exclusion chromatography

To determine the oligomerization state of NrpR, we performed size exclusion chromatography analyses of purified NrpR. A Superdex 200 10/300 Gl column was equilibrated with 20 mM Tris-HCl (pH 8.5), 200 mM NaCl, 1 mM TCEP, and 5% glycerol. The standard curve of molecular weight was obtained using Protein Standard Mix 15–600 kDa (Sigma-Aldrich, Missouri, USA). The mixture contains ribonuclease A (13.7 kDa), ovalbumin (44.3 kDa), gamma globulin (150 kDa), and thyroglobulin (669 kDa). A protein sample was diluted to a concentration of 1 mg/mL in the equilibration buffer and loaded into the column at a flow rate of 0.4 mL/min at room temperature.

### Construction of isogenic mutant strains

Isoallelic strains containing single codon changes were generated as previously described (45). A DNA fragment with approximately 600 bp on either side of the coding region of interest was amplified using the primers listed in Table S2 and cloned into the multi-cloning site of the temperature-sensitive plasmid pJL1055 (46). The resultant plasmids were introduced into *S. salivarius* by competence-based DNA uptake. Briefly, overnight *S. salivarius* growth was diluted in 0.3 mL of chemically defined medium (26) and incubated at 37˚C for 75 min. Subsequently, a synthetic competence-stimulating (ComS) peptide with the amino acid sequence of LPYFAGCL and plasmid was added to the cells and incubated for 3 h at 37˚C. Cells were plated on agar plates containing appropriate antibiotics. Colonies with plasmid incorporated into the *S. salivarius* megaplasmid were selected for subsequent plasmid curing. DNA sequencing was performed to verify the presence of desired mutations and absence of spurious mutations.

### Site-directed mutagenesis

The site-directed mutagenesis was performed to introduce single amino acid substitutions at histidines in the coding region of NrpR. The primers used for mutagenesis are listed in Table S2. The nucleotide sequences of the variants were confirmed by DNA sequencing.

## Data Availability

The coordinates and structure factors for the NrpR structure have been deposited in the Protein Data Bank (PDB) with accession code 9LTH.

## SUPPLEMENTAL MATERIAL

Supplemental tables and figures jb.00059-25-s0001.docx Tables S1 and S2, and Fig. S1 to S4.

ASM does not own the copyrights to Supplemental Material that may be linked to, or accessed through, an article. The authors have granted ASM a non-exclusive, world-wide license to publish the Supplemental Material files. Please contact the corresponding author directly for reuse.
