# Supplementary material for: Environmental pH controls antimicrobial production by human probiotic Streptococcus salivarius
Source: J Bacteriol. 2025 Jun 2;207(6):e00059-25. doi: 10.1128/jb.00059-25 (PMC12186490; doi:10.1128/jb.00059-25)
Supplement: Supplemental tables and figures — Tables S1 and S2, and Fig. S1 to S4. [file jb.00059-25-s0001.docx]

**Supplementary table S1**. Bacterial strains and plasmids used in this study

| **Strain or Plasmid** | **Description** | **Reference** |
| --- | --- | --- |
| **Strains** |  |  |
| WT SAL | *Streptococcus salivarius* K12  ATCC strain number BAA-1024 | ^10^ |
| ∆*nrpR* | Mutant strain that has the *nrpR* (*sarA*) coding region inactivated by marker less deletion in WT SAL | ^3^ |
| *nip** | Mutant strain that has the start codon of *nip* changed to stop codon in WT SAL | ^5^ |
| *nrpR-H9A* | Isoallelic mutant strain that has the alanine substitution at histidine at codon 9 of *nrpR* in WT SAL | This study |
| *nrpR-H15A* | Isoallelic mutant strain that has the alanine substitution at histidine at codon 15 of *nrpR* in WT SAL | This study |
| *nrpR-H52A* | Isoallelic mutant strain that has the alanine substitution at histidine at codon 52 of *nrpR* in WT SAL | This study |
| *nrpR-H144A* | Isoallelic mutant strain that has the alanine substitution at histidine at codon 144 of *nrpR* in WT SAL | This study |
| *nrpR-H281A* | Isoallelic mutant strain that has the alanine substitution at histidine at codon 281 of *nrpR* in WT SAL | This study |
| *E. coli* DH5α | Host strain for cloning purposes |  |
| *E. coli* BL21 (DE3) | Host strain for protein overexpression, *F-, ompT, hsdSB(rB-mB-), gal(λ c I 857, ind1, Sam7, nin5, lacUV-T7 gene1), dcm(DE3)* |  |
| **Plasmids** |  |  |
| *pJL* | Low-copy number plasmid capable of replication in GAS and *Escherichia* coli, Chloramphenicol resistant  Used to generate isoallelic GAS mutants | ^7^ |
| *pET28a* | Overexpression vector for N-terminally hexahistidine tagged recombinant proteins, Km^R^ | Novagen |

**Supplementary table S2**. Primers used in this study

| Primer | Sequence 5’ – 3’ | Purpose |
| --- | --- | --- |
| *nrpR-H9A* top | ACTTTGGCCAGGCAATCGATGCTCTGCGCCGCC | 5’ primer to introduce alanine substitution at histidine 9 of *nrpR* |
| *nrpR-H9A* Bottom | ACTTTGGCCAGGCAATCGATGCTCTGCGCCGCC | 3’ primer to introduce alanine substitution at histidine 9 of *nrpR* |
| *nrpR-H15A* top | GGCGGCGCAGAGCATCGATTGCCTGGCCAAAGT | 5’ primer to introduce alanine substitution at histidine 15 of *nrpR* |
| *nrpR-H15A* Bottom | TTGTTTTACTGAAAAAGCTTTTTGACGTCTAAG | 3’ primer to introduce alanine substitution at histidine 15 of *nrpR* |
| *nrpR-H52A* top | \| TTTTACCTACTGAATGCTCTCAATGTTAAT  GTT \| \| --- \| \|  \| | 5’ primer to introduce alanine substitution at histidine 52 of *nrpR* |
| *nrpR-H52A* Bottom | AACATTAACATTGAGAGCATTCAGTAGGTAAAA | 3’ primer to introduce alanine substitution at histidine 52 of *nrpR* |
| *nrpR-H144A* top | \| ATAGAAACCTGGACTGCCTATGAAACTGTTTTA \| \| --- \| \|  \| | 5’ primer to introduce alanine substitution at histidine 144 of *nrpR* |
| *nrpR-H144A* Bottom | TAAAACAGTTTCATAGGCAGTCCAGGTTTCTAT | 3’ primer to introduce alanine substitution at histidine 144 of *nrpR* |
| *nrpR-H281A* top | \| ATAAAAAATAATCCTGCCATTACATTTAAC \| \| --- \| \|  \| | 5’ primer to introduce alanine substitution at histidine 281 of *nrpR* |
| *nrpR-H281A* Bottom | GTTAAATGTAATGGCAGGATTATTTTTTAT | 3’ primer to introduce alanine substitution at histidine 281 of *nrpR* |
| *tufA* qRTFwd | AAAAACGTCACTACGCTCACAT | 5’ primer for *tufA* qRT-PCR |
| *tufA* qRTRev | GAGCGGCACCAGTGATCAT | 3’ primer for *tufA* qRT-PCR |
| *sarD* qRTFwd | GATGGCAGTTCAAATGGGATTAC | 5’ primer for *sarD* qRT-PCR |
| *sarD* qRTRev | CTGTACCATGACACTCCACAG | 3’ primer for *sarD* qRT-PCR |

Supplementary figure S1

**Supplementary figure S1.** Binding affinity between NrpR and fluoresceinated NIP at different pH conditions was analyzed by fluorescence polarization (FP) assay.

Supplementary figure S2





**Supplementary figure S2.** Oligomerization of NrpR. **A)** The oligomeric state of purified recombinant NrpR was analyzed by size exclusion chromatography (SEC). **B)** The linear fit of the retention volumes of four protein molecular weight standards (Std), Ribonuclease A (13.7 kDa), ovalbumin (44.3 kDa), gamma globulin (150 kDa), and thymoglobulin (669 kDa) to their log molecular weight is shown (black circles). Calculated molecular weight of NrpR (red square) by linear regression analysis is shown. All samples were analyzed in duplicates and at a concentration of 1 mg/ml.

Supplementary figure S3

**Supplementary figure S3.** Analysis of the binding affinity between fluoresceinated NIP and NrpR wild-type or H144A mutant in binding buffer pH 5.5 and 7.5 as assessed by fluorescent polarization assay.

Supplementary figure S4


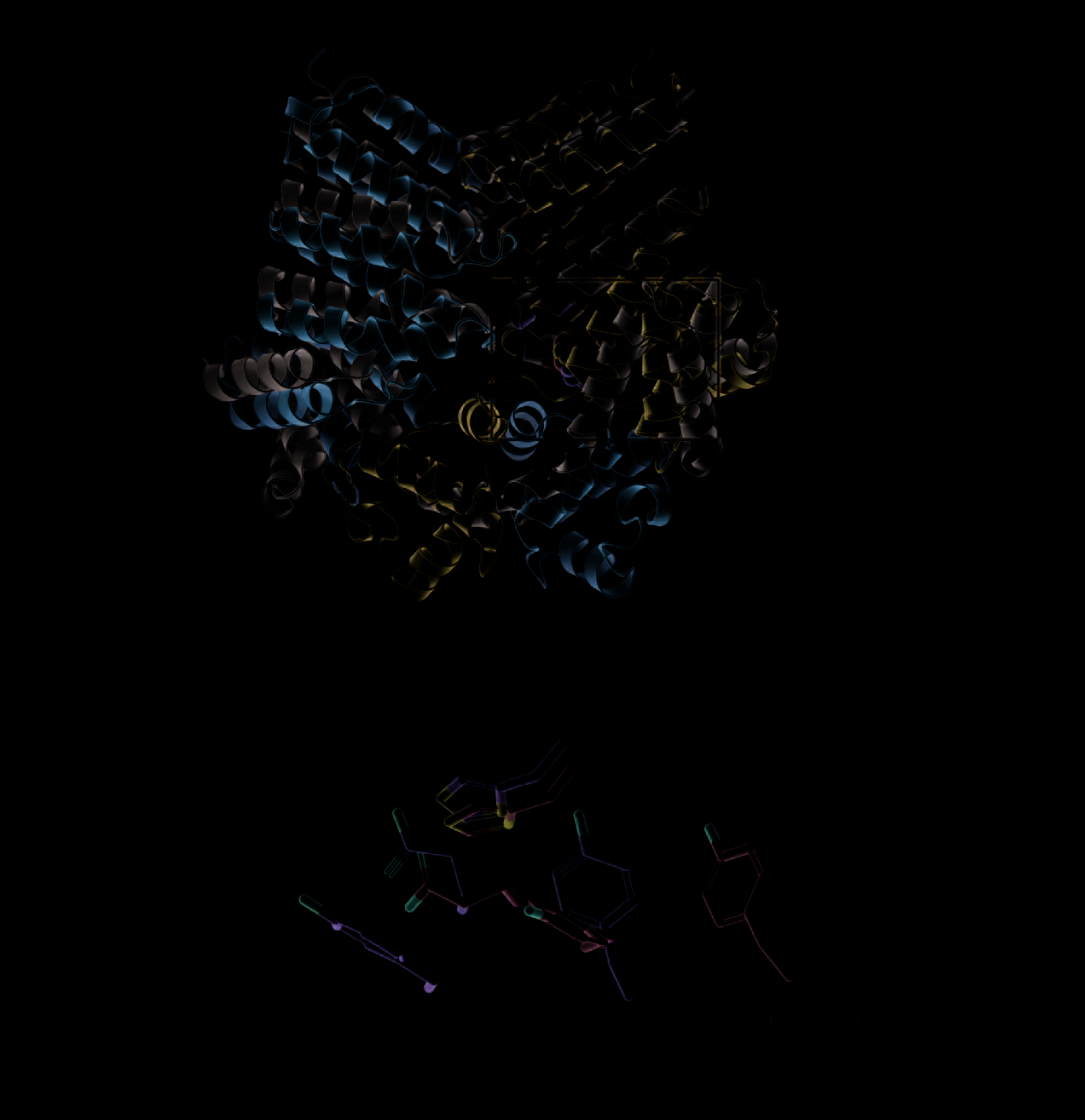


**Supplementary figure S4.** **A)** The overlay of dimeric structures of RopB-CTD (PDB code: 5DL2) and full-length NrpR structures are shown. The secondary structure elements of RopB dimer are colored in light and dark grey. Individual subunits of NrpR dimer are color-coded in blue and orange. The location of H144 in RopB and NrpR structures are highlighted in a black box. **B)** Close up look of similarly positioned H144, Y176, and E185 side chains in RopB and NrpR structures are shown as sticks and labeled. The dissimilar positions of Y182 in RopB and NrpR structures are labeled as Y182-RopB and Y182-NrpR, respectively.
